# A Compact Exposure: Estimating Inhalation of Engineered Nanoparticles in Cosmetic Powders

**DOI:** 10.1289/ehp.120-a245a

**Published:** 2012-06-01

**Authors:** Tanya Tillett

**Affiliations:** Tanya Tillett, MA, of Durham, NC, is a staff writer/editor for *EHP*. She has been on the *EHP* staff since 2000 and has represented the journal at national and international conferences.

The growing use of engineered nanomaterials in consumer products raises questions regarding potential adverse health effects of nano-material exposures. Although a considerable amount of research exists on the toxicity of pure nanomaterials, there is only limited information exposure to nano-materials combined with other ingredients in consumer products. A team of researchers now reports their observations on inhalation exposure through the use of nanotechnology-enabled and non-nanoenabled (“regular”) cosmetic powders [*EHP* 120(6):885–892; Nazarenko et al.].

There are currently more than 1,300 documented nanoenabled consumer products (that is, products that incorporate ingredients meas-uring 1–100 nm in at least one dimension). This figure represents only those products voluntarily reported by manufacturers. In a previous study, these investigators examined the potential for inhalation of nanoparticles through the use of nanoenabled and regular consumer spray products. This time, they analyzed particle size, shape, agglomeration (clustering), and distribution for three nanoenabled and three regular cosmetic powders. To simulate real-world exposures, they applied each powder to a life-size female mannequin head using its enclosed brush or pad. Then they measured the size distribution and concentration of particles that could be inhaled as a result of such application.

**Figure:**
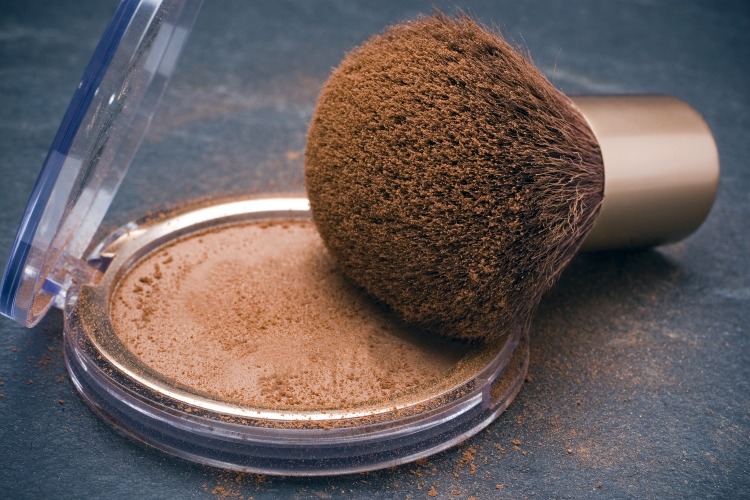
Nanoparticles in cosmetics often cluster into larger agglomerations. © aidaricci/istockphoto.com

All particles in the first sample of nanoenabled powder were in the nanoscale range. The second nanoenabled powder contained a wide range of agglomerated particles, with the majority being nanosized. Despite its label, the third nanoenabled powder contained no nanoscale particles. The researchers did find a number of nanoparticles agglomerated with larger particles in two regular powders, which were not marketed as nanoenabled. The third regular powder contained mostly particles measuring greater than 5 mm in diameter.

Particles under 100 nm in diameter may travel all the way to the terminal alveoli, where air exchange takes place. However, the authors’ electron microscopy data and airborne particle measurements suggest that exposure to nano-particles would be largely through agglomerates of 5–10 mm and larger, which are likely to lodge in the tracheobronchial and head airway regions. But because these agglomerates have a combined surface area greater than that of solid particles of the same size, they may pose different health hazards than solid particles.

The study offers a methodology that could be applied to future studies focusing on real-world inhalation exposures related to nanoenabled consumer products. Results from such studies can help guide estimates of exposures through the short- and long-term use of such products.

